# N-Octanoyl Dopamine, a Non-Hemodyanic Dopamine Derivative, for Cell Protection during Hypothermic Organ Preservation

**DOI:** 10.1371/journal.pone.0009713

**Published:** 2010-03-16

**Authors:** Ralf M. Lösel, Ulf Schnetzke, Paul T. Brinkkoetter, Hui Song, Grietje Beck, Peter Schnuelle, Simone Höger, Martin Wehling, Benito A. Yard

**Affiliations:** 1 Department of Clinical Pharmacology, University Medical Centre Mannheim, Mannheim, Germany; 2 Department of Nephrology, University Medical Centre Mannheim, Mannheim, Germany; 3 Department of Anaesthesiology, University Medical Centre Mannheim, Mannheim, Germany; 4 School of Dentistry, Shandong University, Jinan, People's Republic of China; 5 Department of Medicine, Centre for Molecular Medicine and Cologne Excellence Cluster on Cellular Stress Responses in Aging-Associated Diseases, University of Cologne, Cologne, Germany; University of Cincinnati, United States of America

## Abstract

**Background:**

Although donor dopamine treatment reduces the requirement for post transplantation dialysis in renal transplant recipients, implementation of dopamine in donor management is hampered by its hemodynamic side-effects. Therefore novel dopamine derivatives lacking any hemodynamic actions and yet are more efficacious in protecting tissue from cold preservation injury are warranted. We hypothesized that variation of the molecular structure would yield more efficacious compounds avoid of any hemodynamic effects.

**Methodology/Principal Findings:**

To this end, we assessed protection against cold preservation injury in HUVEC by the attenuation of lactate dehydrogenase (LDH) release. Modification of dopamine by an alkanoyl group increased cellular uptake and significantly improved efficacy of protection. Further variation revealed that only compounds bearing two hydroxy groups in ortho or para position at the benzene nucleus, i.e. strong reductants, were protective. However, other reducing agents like N-acetyl cysteine and ascorbate, or NADPH oxidase inhibition did not prevent cellular injury following cold storage. Unlike dopamine, a prototypic novel compound caused no hemodynamic side-effects.

**Conclusions/Significance:**

In conclusion, we demonstrate that protection against cold preservation injury by catecholamines is exclusively governed by strong reducing capacity and sufficient lipophilicity. The novel dopamine derivatives might be of clinical relevance in donor pre-conditioning as they are completely devoid of hemodynamic action, their increased cellular uptake would reduce time of treatment and therefore also may have a potential use for non-heart beating donors.

## Introduction

Recently a prospective randomized multicenter trial has demonstrated a beneficial effect of donor treatment with low-dose dopamine on immediate kidney graft function [1]. Kidney transplant recipients who received a graft from a dopamine treated donor had a significantly decreased need for dialysis after kidney transplantation compared to the untreated control group. The salutary effect of dopamine was more pronounced when cold ischemia time was prolonged and translated in a better graft survival in this sub-group. In view of the protective effect of dopamine reported in prospective and retrospective clinical studies [1], [2], [3], [4] and based on animal studies [5], [6] as well as *in vitro* experiments [7], [8], [9], current evidence suggest that dopamine has the propensity to protect allografts from the deleterious event of cold ischemia [10], [11]. Implementation of low dose dopamine in donor management would therefore be genuine rationale for maintaining organ quality even after prolonged cold storage. The caveat however is that in brain-dead donors catecholamine clearance is changed and hence low dose dopamine treatment might result in tachycardia and hypertension in approximately 15% of the brain-dead donors [12]. In addition, the duration of dopamine treatment is at present not known. Nonetheless there is a significant relation between the time of dopamine treatment and efficacy on preventing delayed graft function [1]. This might be explained by the fact that dopamine is rapidly degraded in the circulation by monamine oxidase, hence sufficient tissue dopamine levels can only be obtained by increasing the treatment dose or time of dopamine treatment. The former cannot be used in brain dead donors as this would increase the incidence of tachycardia and hypertension.

The use of non heart beating donors is world-wide increasing. Yet, the incidence of delayed graft function is significantly increased when renal allografts from such donors are used [13], [14], [15]. Since dopamine treatment in these donors can only be initiated after cardiac arrest, and the time required for obtaining sufficient tissue dopamine levels might take several hours, dopamine treatment of non heart beating donors is not an option to reduce the incidence of delayed graft function.

Given these limitations of dopamine, there is an unmet need for compounds that lack hemodynamic action and yet are more efficacious than dopamine. To meet this objective, we delineated the structural entities within catecholamine mimetic compounds that convey protection against hypothermic injury by systematic variation of their molecular structure. We aimed to synthesize compounds devoid of any hemodynamic side effects that protect endothelial cells against cold preservation injury more potently.

## Results

### Protection against hypothermic injury depends on lipophilicity and redox activity

**Figure 1:**
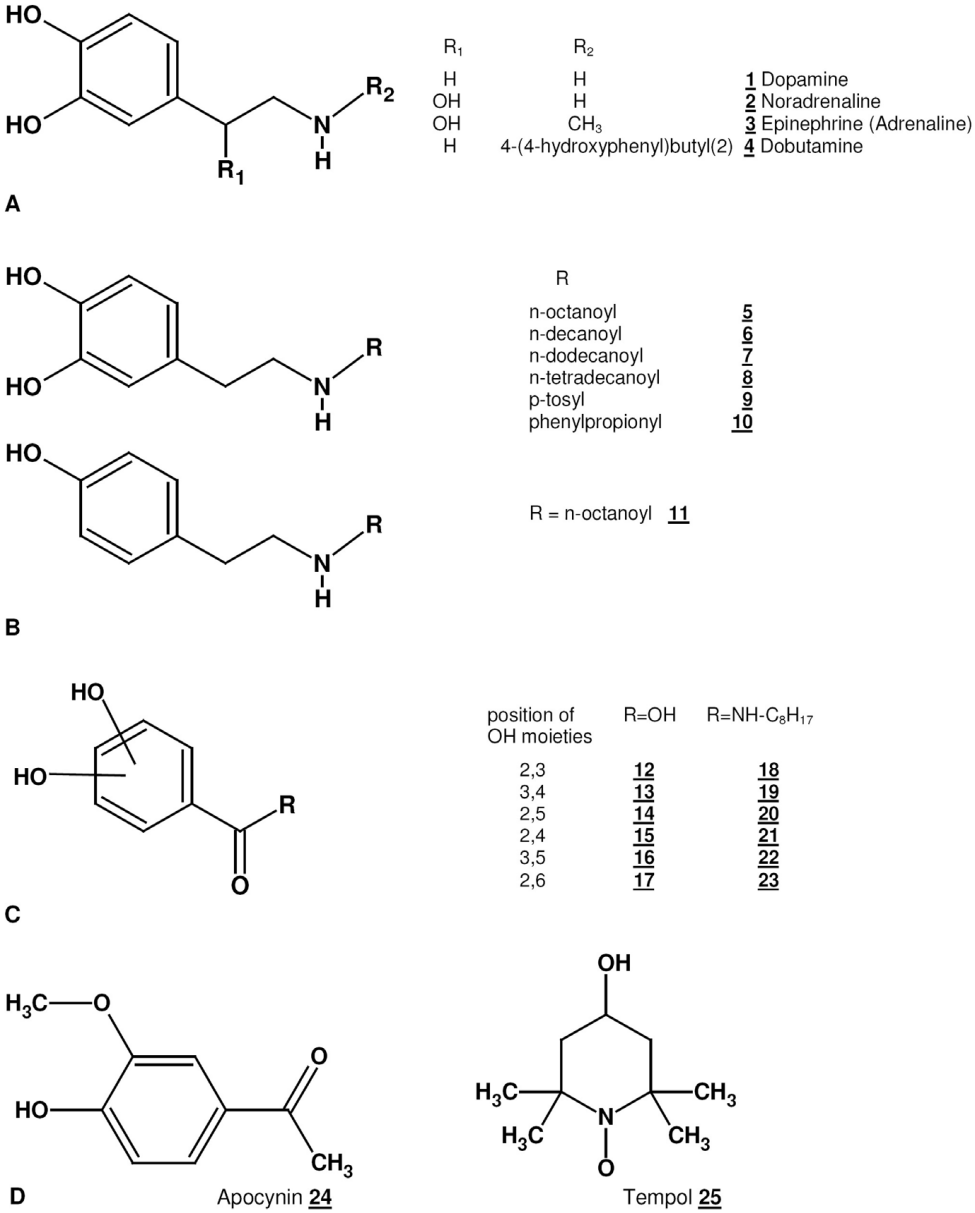
Molecular structures of compounds used in this study. (A) Catecholamines previously reported to exert a protective effect on endothelial cells against cold damage. (B) Structurally related derivatives of dopamine synthesized and used in this work. (C) Chemically possible isomers of the dihydroxybenzoyl fragment used as free acids (R  =  OH) or as much more lipophilic n-octyl amides (R  = NH-C_8_H_17_). The compounds with hydroxy groups in ortho or para position (2,3; 3,4 or 2,5) present with strong reducing capacities, while the other isomers are much weaker reducing agents. (D) Structure of the chemically unrelated substances apocynin, a NAPDH oxidase inhibitor, and tempol (2,2,6,6-tetramethylpiperidine-1-oxyl), a stable radical often employed as a low molecular weight superoxide dismutase mimetic.

**Figure 2:**
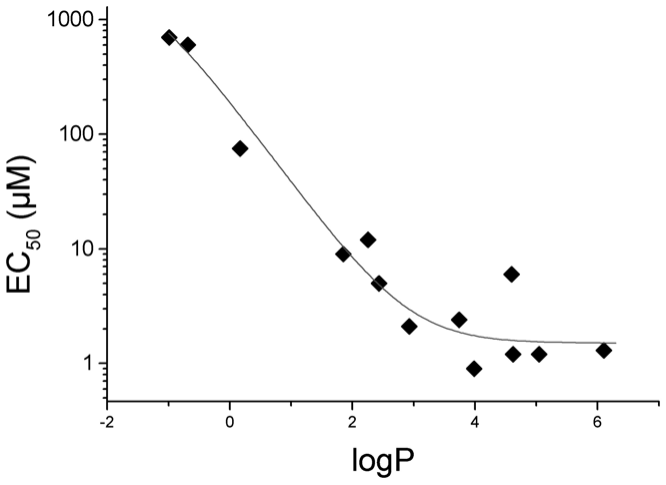
EC50 and logP values follow a sigmoid Boltzmann distribution. EC50 values expressed as mean (n = 3) are plotted against calculated logP values for the reducing compounds (1–10 and 18–20) listed in table 1. A sigmoid (Boltzmann) line was found to fit the data set with A1 = 1.22, A2 = 1000, x0 = −0.67, dx = −0.58.

New compounds were synthesized as described in the method section and tested for their protective effects against hypothermic injury. To this end HUVEC were pre-incubated for 2 hrs and EC50 values were calculated for each substance based on the LDH release after 24 hrs cold storage at 4°C (**Table 1**). Modification of dopamine by an alkanoyl group (**Figure 1**, compounds 5–8) increased logP values and was associated with increased efficacy as indicated by a 40-fold decrease in EC50 values compared to dopamine. Among the protective compounds, the EC50 values correlated with their calculated logP values (**figure 2**). These results suggest that the more lipophilic a compound is the more potently it protects against hypothermic injury up to a logP value of 2.5.

**Table 1:** Chemical characteristics of compounds used in this study.

Compound #	Fig	EC50 [µM]	strong reducing agent?	logP (calc.)
1	1A	∼75	+	0.169*
2	1A	>100	+	−0.989*
3	1A	>100	+	−0.685*
4	1A	5	+	2.433*
5	1B	2.1±0.2	+	2.929
6	1B	0.9±0.2	+	3.987
7	1B	1.2±0.2	+	5.045
8	1B	1.3±0.2	+	6.103
9	1B	12±1	+	2.253
10	1B	9±1	+	1.852
11	1B	>100	−	3.526
12	2	>100	+	1.622
13	2	>100	+	1.062
14	2	>100	+	1.622
15	2	>100	−	1.622
16	2	>100	−	0.992
17	2	>100	−	2.252
18	2	1.2±0.1	+	4.621
19	2	2.4±0.2	+	3.741
20	2	6±1	+	4.6
21	2	>100	−	4.621
22	2	>100	−	3.671
23	2	∼90	−	3.741

The protective effect of various compounds against cold-induced endothelial cell injury expressed as the concentration required to inhibit 50% LDH release and lipophilicity expressed as logarithm of the partition coefficient (logP, *denotes experimental values taken from public sources). Depicted are mean values ± SD (n = 3).

In addition to its increased lipophilicity, dobutamine (compound 4), which is characterized by an EC50 value of 5 µM, contains a second aromatic residue. Therefore, we next addressed the question whether 2 aromatic residues would further increase the protective capacities of these compounds. Compounds 9 and 10 showed decreased EC50 values compared to dopamine (compound 1) but were less effective than the chemically related dobutamine (compound 4) despite having comparable logP values. These data demonstrate that the protective effect of compounds with 2 aromatic residues is not superior to those with 1 aromatic residue only.

Removal of the 3-OH group from the highly protective compound 5 yielding compound 11 further increased lipophilicity (logP 3.526 vs. 2.929) but abolished the protective effect (EC50 >100 µM vs. 2.1 µM±0.2). This could indicate that either a strongly reducing catechol function is required for maximal protection, or suggest a receptor mediated phenomenon that recognizes the intact dopamine fragment only. To test the hypothesis that the protective effect of catechol compounds depends on its reducing capacity and is independent of the dopamine structure, we synthesized the octylamide derivates (compounds 18–23) of all possible dihydroxy benzoic acids (compounds 12–17), including 3 reducing (compounds 12–14 and 18–20) and 3 non (or weakly) reducing (compounds 15–17 and 21–23) structures. While the free acids (compounds 12–17) were all ineffective (EC50 >100 µM), the reducing octylamides (compounds 18–20) revealed EC50 values between 1.2 and 6 µM. In addition, all non-reducing octylamides (compounds 21–23) were ineffective despite comparable logP values.

Taken together, all compounds identified as protective cover diverse structures at the benzene nucleus and share only one common property, i.e. their reducing capacity. Therefore, it is unlikely that a specific receptor-mediated process underlies the protective properties of catecholamines and related substances.

Subsequently, to investigate whether the protective properties of these compounds reflect a general anti-oxidative effect, we also employed additional reducing compounds and specific inhibitors of oxidative pathways. Both ascorbate and N-acetyl cysteine were not protective against hypothermic injury when used up to 300 µM (data not shown). Similarly, increasing doses up to 3 mM of apocynin, an inhibitor of the NAPDH oxidase or up to 300 µM of tempol (2,2,6,6-tetramethylpiperidin-n-oxyl), a scavenger of reactive oxygen species, did not confer cellular protection (data not shown).

### N-acylation of dopamine abolishes the hemodynamic effects in vivo

**Figure 3:**
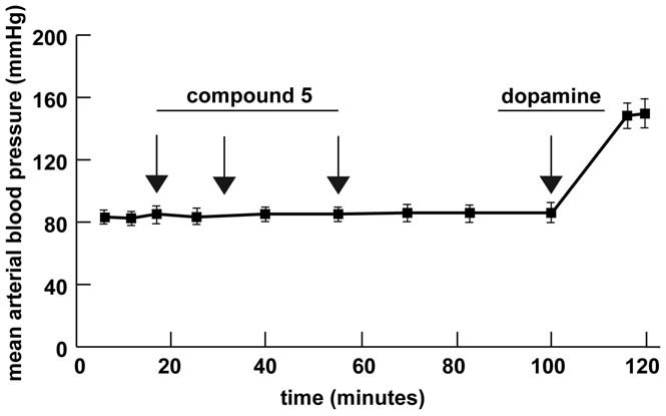
Hemodynamic activity of N-octanoyl-dopamine (compound 5). Mean arterial blood pressure was measured online in anaesthetized rats while infusing equimolar concentrations of compound 5 (18 µg/kg/min) or dopamine (10 µg/kg/min). Depicted are mean values ± SD (n = 3).

We next sought to test potential hemodynamic side effects of the highly protective compound 5 *in vivo*. To this end, male Fisher rats received 3 consecutive intravenous injections of N-octanoyl-dopamine (0.05 µmoles/kg body weight) and systemic blood pressure was continuously monitored by an arterial catheter. As expected the loss of charge at nitrogen in N-octanoyl dopamine (compound 5) abolished any effect on mean arterial blood pressure (**figure 3**). To exclude any potential competitive inhibition of dopaminergic receptors by compound 5, a single dose of dopamine (0.05 µmoles/kg body weight) was administered 100 minutes after the initial N-octanoyl dopamine injection. Animals responded with a significant increase in mean arterial blood pressure within 20 minutes (83 mmHg ± 4 vs. 162 mmHg ± 6). Thus, N-acylation of dopamine impairs receptor binding and does not yield dopamine antagonistic products.

### Increased lipophilicity enhances cellular uptake

**Figure 4:**
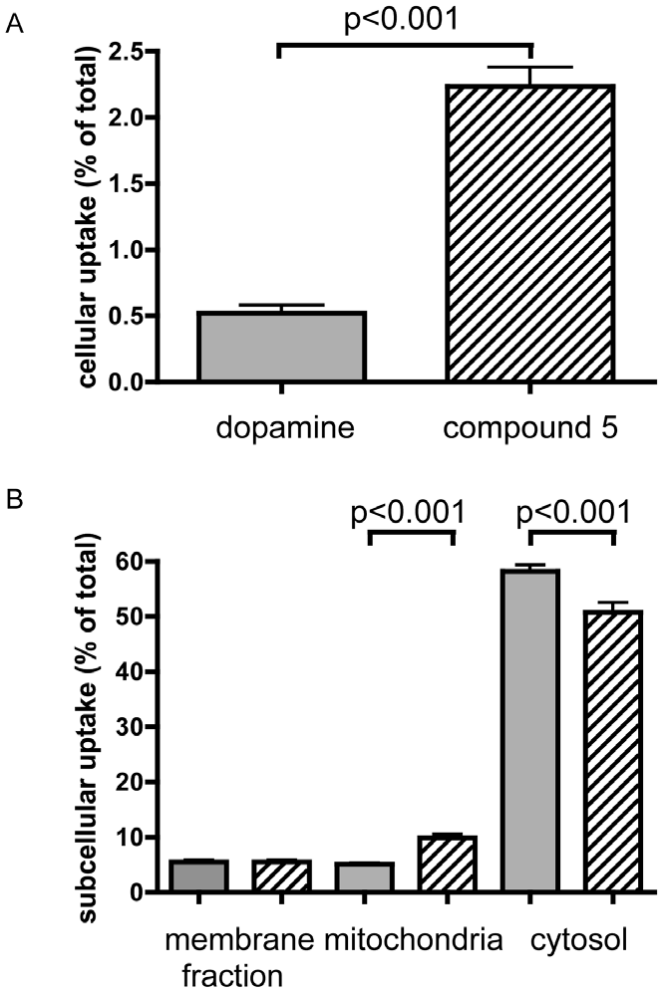
Subcellular distribution of radioactivity from N-Octanoyl dopamine or dopamine. HUVEC were incubated for 2 hrs with equal amounts of [^3^H]-octanoyl-dopamine (compound 5) or 7,8-[^3^H]-dopamine. Results are expressed as mean ± SD (n = 3) incorporation relative to the amount of radioactivity applied. (A) Total cellular uptake of [^3^H]-octanoyl-dopamine (compound 5) or 7,8-[^3^H]-dopamine relative to total amount applied. (B) Subcellular distribution of radioactivity from [^3^H]-octanoyl-dopamine (compound 5, hatched bars) or 7,8-[^3^H]-dopamine (grey bars), relative to total radioactivity uptake.

Hypothermia leads to a redox imbalance with a relative increase in ROS. Mitochondrial function and the generation of ATP necessary for the generation of redox equivalents are greatly impaired. Recently, we have shown that dopamine preserves mitochondrial function during cold storage in HUVEC [7], [8]. Therefore, we tested the hypothesis that N-acylation of dopamine would increase cellular and mitochondrial uptake which would contribute to the increased protective effects of these compounds. To this end we synthesized [^3^H] labelled compound 5 from 7,8-[^3^H] dopamine and studied its subcellular distribution after 2 hrs incubation at 37°C. Based on radioactivity, total uptake of compound 5 was approximately 4-fold greater compared to dopamine when employed at the same concentration (**figure 4A**). Increased cellular uptake was also accompanied by increased relative uptake in the mitochondria and a decrease in the cytosolic compartment while the membrane fraction showed comparable levels of [^3^H] (**figure 4B**). In mitochondria, the *relative* concentration of compound 5 was increased about 2-fold, corresponding to approximately 8 times (in absolute values) more radioactivity in the mitochondria compared to dopamine.

## Discussion

Novel, experimental strategies to further reduce pre-transplantation injury include treatment of brain-dead heart-beating donors prior to organ explantation. Pre-treatment with catecholamines is positively associated with improved organ function following transplantation [1], [2], [3], [4]. The aim of this study was to elucidate the structural entities of catecholamines and related substances that confer protection against hypothermic injury.

The first major finding of this study was that the single common feature of all protective compounds tested was the presence of 2 hydroxy groups at the benzene nucleus, located either in ortho or in para position. Additional substituents were of minor importance. Ortho and para isomers are known to be strong reducing agents due to the ease of quinone formation, which cannot occur in meta isomers. The reducing characteristics are in line with previous findings where the loss of its reducing capacity by oxidation abolished the protective effect of dopamine [9].

In addition, sufficient lipophilicity was required for catecholamines to protect against hypothermic injury. As expected, N-acylated dopamine derivatives entered the cells more easily than unmodified dopamine and accumulated to a greater proportion in the mitochondrial compartment. Although the calculated logP values may differ from experimentally determined values, the presented numbers show significant agreement with previously published data which are available for some compounds. As logP refers to uncharged compounds, additional effects cannot be excluded. In addition, dihydroxybenzoic acids are present in their ionized forms at physiological pH, which may influence their cellular uptake and thus protective potency. Within the series of uncharged amide compounds, the assumed correlation of logP with their respective protective effects appears to be valid. The aryl derivates (compound 9+10) exhibit a slightly lower efficacy as expected from their logP value. However, in contrast to catechol derivates with comparable logP values they lack an extended alkyl chain that would enable them to insert into lipid membranes more easily.

Mitochondria might display a relevant target for protection by catecholamines [7], [8]. Several lines of evidence support this hypothesis. First, hypothermia reduces respiratory chain activity resulting in depletion of ATP and reduction equivalents, leading to a redox imbalance with a relative increase in ROS [8]. Catecholamines present with strong reducing characteristics and could prevent, at least in parts, mitochondrial injury caused by increased ROS. Second, increased ATP consumption causes a breakdown in mitochondrial membrane potential leading to calcium accumulation [16], which further aggravates mitochondrial dysfunction. Dopamine has been shown to delay these effects in HUVEC during cold storage [7], [8].

A third major finding was that the protective effects of catecholamines are independent of any receptor-mediated process. Highly specific structural entities are required for a ligand to interact with its specific receptor, which can be expected to respond much more selectively to significant molecular modifications. Therefore, the variety of dihydroxyphenyl structures that present with protective characteristics strongly disfavours any involvement of a specific receptor mediated process. We also tested and excluded that N-octanoyl-dopamine (compound 5) exhibits any hemodynamic effects *in vivo* and does not function as a competitive receptor inhibitor either. These results extend previous reports where we demonstrated that the protective effect of dopamine is independent of *de novo* protein synthesis. Interestingly, a relative short incubation time up to 30 minutes is sufficient to achieve maximal protection (data not shown). Taken together, these results prove that the protective effect of catecholamines lies within their molecular moieties.

Based on their chemical structure catechols are also able to form complexes with free iron ions. Several studies reported an increase in the chelatable iron pool during hypothermic preservation which further contributes to cellular injury [17], [18], [19]. On the basis of our findings, we can exclude a significant contribution of catecholamines and their capacity to complex iron in relation to their protective effect for the following reason. In most studies on mixed ligand complexes of iron, catechol yields the most stable complexes. While hydroxy groups at the benzene nucleus in meta and para position, i.e. resorcinol and hydroquinone (or their functional derivatives) usually result in equally stable iron complexes, they yield much less stable iron complexes than catechol (ortho) compounds. In our study, compounds with para- or ortho-dihydroxy groups were equally protective against cold preservation injury, while the meta derivatives were not protective. Thus, there was no correlation between the capacity of catechols to protect against hypothermic injury and their expected ability to form iron complexes.

Based on the structural requirements we hypothesize that insertion of the compounds into lipid compartments might prevent oxidative damage at or near hydrophobic compartments. Regardless of the mechanism, an efficient protective substance requires an ortho or para dihydroxybenzene partial structure, is preferably uncharged with an alkyl chain and exhibits an overall lipophilicity exceeding a logP value of 2.5.

In conclusion, we have demonstrated that a strong reducing capacity by suitable substituents at the benzene nucleus as well as sufficient lipophilicity mediate the protective effect of catecholamines against hypothermic injury. Dihydroxy phenolic substances that lack any hemodynamic effects can also fulfil these structural requirements. Hence, these novel compounds might be considered for donor pre-conditioning to protect allografts against cold preservation injury without influencing blood pressure in the donor. As cellular uptake is significantly increased, the use of these novel compounds would decrease the time required for treatment and, therefore, offer a mean to reduce the incidence of delayed graft function when renal allograft of non heart beating donors are used.

## Methods

### Cell isolation and culture

Human umbilical vein endothelial cells (HUVEC) were isolated as described previously and cultured in endothelial cell growth medium (Promocell Heidelberg, Germany) in T25 flasks (Greiner, Frickenhausen, Germany) coated with 1% gelatine (Fluka, Neu-Ulm, Germany).

### Hypothermic preservation injury

Hypothermic preservation injury of HUVEC was assessed by lactate dehydrogenase (LDH) release. LDH assays were performed using a commercial system as recommended by the manufacturer (Roche Diagnostics, Mannheim, Germany). Briefly, HUVEC were seeded in 24-well plates (Greiner), grown until confluence and preincubated with test substances for 2 hrs. The plates were washed three times with 1 ml of PBS and stored for 24 hrs at 4°C in phenol red free medium (PAA, Pasching, Austria). 100 µl aliquots of supernatants were used to determine LDH release with phenol red free medium as blank control. Experiments were performed in triplicate (n = 3) and results are expressed as OD at 490 nm corrected for the blank.

### Synthesis of compounds

Test compounds were synthesized from commercially available precursors (all from Fluka unless stated otherwise) and were purified by recrystallization to homogeneity as demonstrated by thin layer chromatography (TLC). Samples investigated by NMR (Bruker AC250) yielded spectra in accordance with the expected structures. LogP values were calculated using the engine at www.daylight.com/daycgi/clogp or at www.molinspirations.com. All compounds synthesized in this study are shown in **Table 1**.

### General procedure for acylated dopamine derivates

Carboxylic acids were converted to their mixed anhydride derivatives by reaction with ethyl chloroformate and the mixed anhydride incubated with dopamine dissolved in dimethyl formamide (DMF) at 50 mg/ml, in the presence of N-ethyl diisopropylamine. Ethyl acetate was added to the mixture, and after washing with sodium hydrogen carbonate and diluted sulphuric acid, evaporation of the solvent yielded the acylated product.

### General procedure for dihydroxybenzoyl amides

Stoichiometric amounts of dihydroxybenzoic acid, amine and dicyclohexyl carbodiimide were dissolved in THF and kept overnight at room temperature. After filtration, the solution was evaporated, re-dissolved in ethyl acetate and washed sequentially with diluted acid, brine, and sodium bicarbonate. After drying, the solvent was evaporated.

### [^3^H]-N-octanoyl dopamine (compound 5)

[^3^H]-N-Octanoyl dopamine was synthesized from 1.85 MBq 7,8-[^3^H]-dopamine (Amersham, Freiburg, Germany). About 65% of radioactivity was recovered. TLC, spraying with En^3^hance (Perkin Elmer, Waltham, MA) and 7d exposure on X-ray film at −80°C revealed >90% purity as determined by comigration of nonradioactive N-octanoyl-dopamine.

### Tracer studies and subcellular fractionation

HUVEC cultured to confluence in T175 flasks (Greiner) were incubated with 925 kBq (25 µCi) of [^3^H]-octanoyl-dopamine or [^3^H]-dopamine for 2 hrs. Hereafter the cells were washed and a subcellular fractionation was performed as recommended by the manufacturer (Geno Technology, St. Louis, MO). Briefly, cell lysates were prepared by homogenization were centrifuged; the supernatants were further centrifuged for 2 min at 4,000 x *g*. Cytosolic proteins and cellular organelles were present in the supernatant (S1). The pellet (P1) contained cell membranes and the nuclear fraction. S1 was further centrifuged (20 min, 10,000 x g) to separate cellular organelles (pellet P2) from cytosolic proteins (supernatant S2). P2 was subjected to hypotonic shock followed by centrifugation (20 min 10,000 x *g*) to separate mitochondria (pellet P3) from lysosomes (supernatant S3). Supernatant S2 was further centrifuged for 1 h at 100,000 x *g*. Purity of the subcellular fractions was tested by Western blotting. Radioactivity was measured in aliquots collected directly after douncing and in aliquots of P1, P3 and S2. Samples were mixed with 3 ml scintillation cocktail and counted in a Beckman LS6000.

### Measurements of hemodynamic parameters

Male Fisher rats (250 g) were anaesthetised with ketamine and xylazine. Dopamine or N-octanoyl-dopamine (0.05 µmoles/kg body weight) was infused into the femoral vein. Systemic blood pressure (mean arterial pressure, mmHg) was continuously measured by a femoral arterial catheter (SIMS Portex, UK). All procedures were performed according to the guidelines of the American Physiological Society and were approved by the local authorities (Regierungspraesidium Karlsruhe AZ 35-9185.81/G-61/05).
